# Nongenotoxic ABCB1 activator tetraphenylphosphonium can contribute to doxorubicin resistance in MX-1 breast cancer cell line

**DOI:** 10.1038/s41598-021-86120-6

**Published:** 2021-03-22

**Authors:** Raimonda Kubiliute, Indre Januskeviciene, Ruta Urbanaviciute, Kristina Daniunaite, Monika Drobniene, Valerijus Ostapenko, Rimantas Daugelavicius, Sonata Jarmalaite

**Affiliations:** 1 Institute of Biosciences, Life Sciences Center, Vilnius University, 10257 Vilnius, Lithuania; 2 Department of Biochemistry, Faculty of Natural Sciences, Vytautas Magnus University, 44404 Kaunas, Lithuania; 3 National Cancer Institute, Santariskiu 1, 08406 Vilnius, Lithuania

**Keywords:** Cancer, Cell biology, Genetics, Molecular biology

## Abstract

Hyperactivation of ABC transporter ABCB1 and induction of epithelial–mesenchymal transition (EMT) are the most common mechanism of acquired cancer chemoresistance. This study describes possible mechanisms, that might contribute to upregulation of *ABCB1* and synergistically boost the acquisition of doxorubicin (DOX) resistance in breast cancer MX-1 cell line. DOX resistance in MX-1 cell line was induced by a stepwise increase of drug concentration or by pretreatment of cells with an ABCB1 transporter activator tetraphenylphosphonium (TPP^+^) followed by DOX exposure. Transcriptome analysis of derived cells was performed by human gene expression microarrays and by quantitative PCR. Genetic and epigenetic mechanisms of ABCB1 regulation were evaluated by pyrosequencing and gene copy number variation analysis. Gradual activation of canonical EMT transcription factors with later activation of *ABCB1* at the transcript level was observed in DOX-only treated cells, while TPP^+^ exposure induced considerable activation of ABCB1 at both, mRNA and protein level. The changes in ABCB1 mRNA and protein level were related to the promoter DNA hypomethylation and the increase in gene copy number. ABCB1-active cells were highly resistant to DOX and showed morphological and molecular features of EMT. The study suggests that nongenotoxic ABCB1 inducer can possibly accelerate development of DOX resistance.

## Introduction

Despite significant advances in cancer diagnosis and treatment, resistance to chemotherapy remains an important barrier to successful cancer therapy^1^. Multidrug resistance, known as the simultaneous resistance to different classes of drugs, is the leading cause of cancer recurrence and lethal outcomes^2^. An increased active efflux of drugs, mediated by ATP binding cassette superfamily proteins (ABC transporters) is the most common mechanism by which cancer cells develop resistance to various chemotherapeutics^3^. ABC transporters are ubiquitous and their heterogenous expression is observed in various body tissues, where they perform a detoxification of host cells and protect the body against xenobiotics^4^. In cancer, an increased expression level of these transporters leads to chemotherapy resistance due to their ability to extrude from the cells a number of drugs^4^. The overexpression of ABCB1 (P-glycoprotein), one of the main members of the ABC transporter family encoded by the *ABCB1* gene, can be triggered by a myriad of factors such as antibiotics, analgesics^5^, retinoic acid, sodium butyrate, UV irradiation^6^, radiotherapy^7^, and various certain chemotherapeutic anticancer drugs^3^. Together with the drug efflux, increased expression of ABC transporters has been correlated with the avoidance of apoptosis, cell migration, invasion and metastasis^8,9^, altogether resulting in tumor aggressiveness.

Drug resistance can be either inherent or acquired during the treatment, but most possibly the interaction of both mechanisms drives the rapid development of treatment refractory cancer^10^. Intrinsic drug resistance is quite widespread among tumors of several localizations due to the inherently high expression of ABC transporters in the healthy tissues, while in other cases noncarcinogenic compounds, like vitamins D, curcuminoids, or flavonoids, can stimulate their activity^11,12^. Besides, in tumors, a subpopulation of self-renewing cells called cancer stem-cells (CSCs) have been shown to express higher levels of drug transporters allowing them to survive chemotherapy and give rise to tumor mass, predominantly composed of therapy-resistant cancer cells^8^. Beside direct extrusion of various drugs, the hyperactivation of ABC transporters also participate in the development of chemoresistance through activation of epithelial-to-mesenchymal transition (EMT) phenomena^2,13–15^. Though the mechanisms of intrinsic and extrinsic chemoresistance are widely studied, the interaction between both these pathways remains poorly perceived.

One of the potential cause for the increased expression of *ABCB1* is gene amplification, which was found in chemoresistant cells^13,14^. Other notable mechanisms that are possibly responsible for *ABCB1* upregulation are epigenetic alterations, especially DNA methylation, that involve *ABCB1* promoters. The *ABCB1* gene has been reported to embrace two distinct upstream and downstream promoters^16^. The latter one functions as the major promoter in various *ABCB1* expressing cell lines and tissues^17^. Furthermore, it was shown that hypomethylation of *ABCB1* downstream promoter is associated with increased *ABCB1* expression and acquisition of multidrug resistance in breast cancer cells^13,14^.

Breast cancer is a leading cause of cancer-related deaths among women in the Western world. Anthracyclines, including doxorubicin (DOX), along with taxanes, cyclophosphamide and platinum compounds are the main chemotherapeutic agents applied for breast cancer treatment. However, chemoresistance is the major cause of the disease progression following chemotherapy. In the present study, the mechanisms of acquired cellular chemoresistance were studied in breast cancer cell line MX-1 exposed to gradually increasing concentration of the chemotherapeutic compound DOX. Nongenotoxic phosphorganic compound tetraphenylphosphonium cation (TPP^+^)—a potent substrate and activator for ABCB1 transporter^18^—was used in the cell line model of intrinsic chemoresistance. Finally, DOX highly resistant cell subline was derived from ABCB1-activated, TPP^+^ pretreated cells. Chemoresistance in all cell sublines was closely associated with the EMT, and the ABCB1 hyperexpression was a possibly trigger of this process.

## Results

### Generation of DOX-resistant MX-1 cell lines

**Figure 1:**
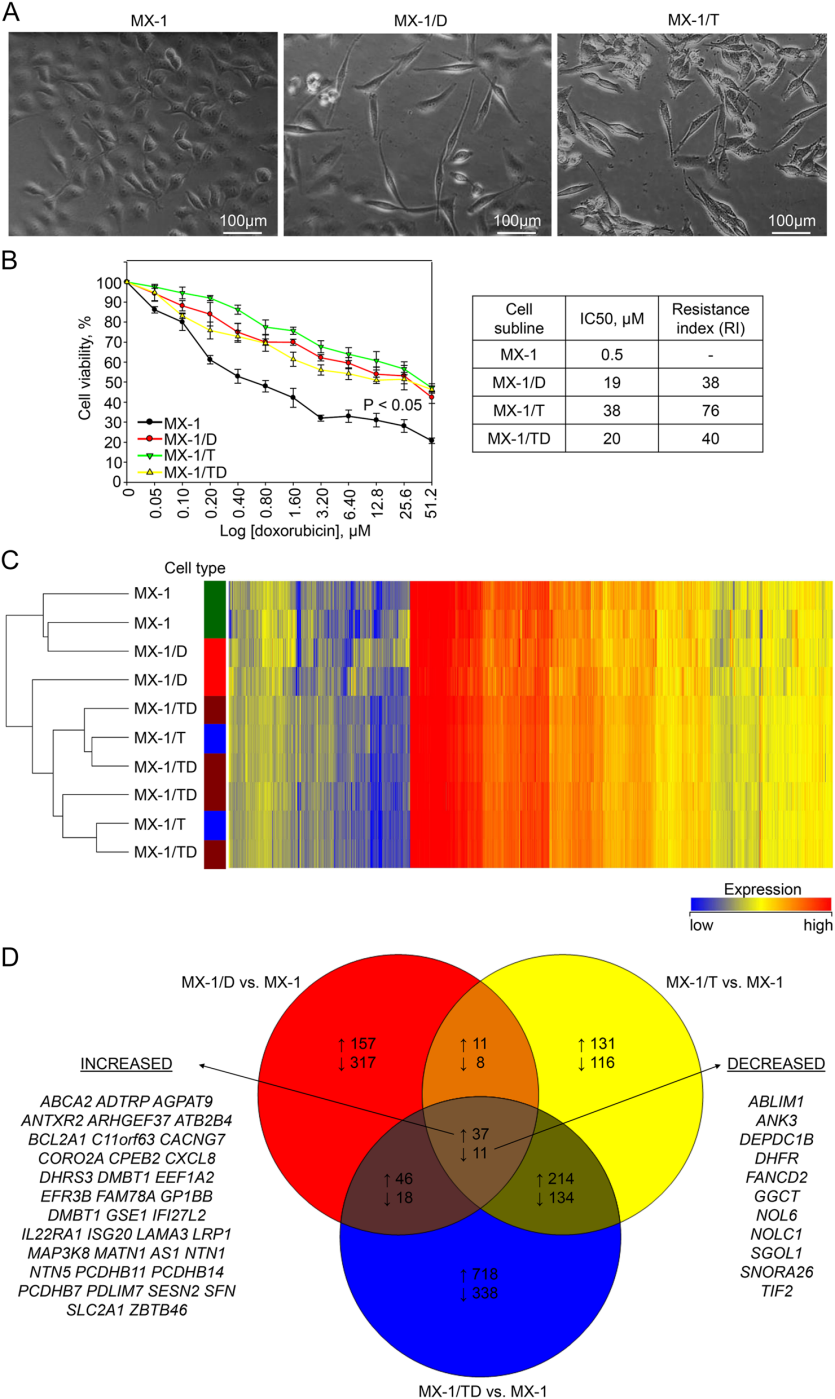
Characterization of chemoresistant MX-1 cell sublines. (**A**) Representative phase-contrast microscopic images demonstrating morphology of MX-1 cells and morphological changes in MX-1/D and MX-1/T cells cultured in media with doxorubicin and TPP^+^ respectively. Magnification—× 200. (**B**) Cytotoxicity of doxorubicin measurements with the MTT assay in the parental MX-1 cells and chemoresistant MX-1/D (cultured with 80 nM of doxorubicin), MX-1/T (cultured with ABCB1 transporter activator TPP^+^ (128 nM)) and MX-1/TD (MX-1/T cells cultured in media supplemented with up to 1280 nM of doxorubicin for a week). The indicated P-value was calculated at the end point of the experiment, compared the viability of each chemoresistant cell sublines with wild-type cells. Resistance index was calculated IC50 values of chemoresistant cells dividing by IC50 of parental MX-1 cells. (**C**) Heatmap showing the gene expression profile of the parental MX-1 and chemoresistant MX-1/D, MX-1/T and MX-1/TD cells. The results were obtained by Human Gene Expression (v2) 8 × 60 K microarrays (design ID 039494). (**D**) Venn diagram, showing the numbers of overlapping deregulated genes among MX-1/D, MX-1/T and MX-1/TD sublines compared to untreated MX-1 cells. Listed genes are common in the all investigated cells.

A series of DOX-resistant MX-1 cell sublines were established during the selection of parental cells for survival in the media with increasing concentrations (10 to 80 nM) of DOX. Cytotoxicity measurement with the MTT assay revealed a 38-fold increased resistance to DOX in MX-1/D80 cells as compared to the parental cell line. The IC50s in parental MX-1 cells and MX-1/D80 were 0.5 µM and 19 µM respectively (Fig. 1B). Development of DOX-resistance was accompanied by the evident morphology changes from cobblestone-like with relatively strong cell–cell adhesion to a spindle-like appearance (Fig. 1A).

Followed by repeated treatments of parental cells with increasing concentrations (up to 128 µM) of TPP^+^, cell subline MX-1/T was obtained. In the cell survival assay, IC50 of DOX in MX-1/T cells was 38 µM (Fig. 1B) and the resistance index has reached 76. Moreover, MX-1/T cells acquired resistance to other chemotherapeutics, particularly paclitaxel and cisplatin, as well (Additional file 1, Fig. 1). Similarly, to MX-1/D, MX-1/T cells became elongated in shape and were dissociated from surrounding cells (Fig. 1A). These TPP^+^ pretreated cells was exposed to 1280 nM of DOX for a week, and MX-1/TD cell subline was generated which was able to maintain the similar resistance index (40) as in the case of MX-1/D80 cells.

### Different mRNA expression pattern in chemoresistant MX-1 cells

For the characterization of the mechanisms of chemoresistance, the microarray-based genome-wide gene expression analysis of MX-1/D, MX-1/T and MX-1/TD, as well as parental MX-1 cells, was performed. Comparison of gene expression between the parental cells and their chemoresistant derivatives revealed numerous changes in gene expression. Cluster analysis showed that TPP^+^-treated cells (with or without DOX exposure), tended to group separately from the parental cell line or DOX-only treated cells (Fig. 1C).

A total of 605, 662 and 1516 genes were significantly deregulated (FC ≥ 2, *P* < 0.05) in MX-1/D, MX-1/T, and MX-1/TD cells, respectively, as compared to parental MX-1 cells (Fig. 1D). All chemoresistant cell sublines shared 37 up- and 11 downregulated genes (Fig. 1D). MX-1/T and MX-1/TD had the largest number of overlapping genes with deregulated expression (251 up- and 145 downregulated genes). The most abundant differences in gene expression (457 up- and 508 downregulated) was identified between MX-1/D and MX-1/TD cells, and the number of overlapping deregulated genes was small (Fig. 1D).

### Cellular responses deregulated in chemoresistant cell sublines

**Figure 2:**
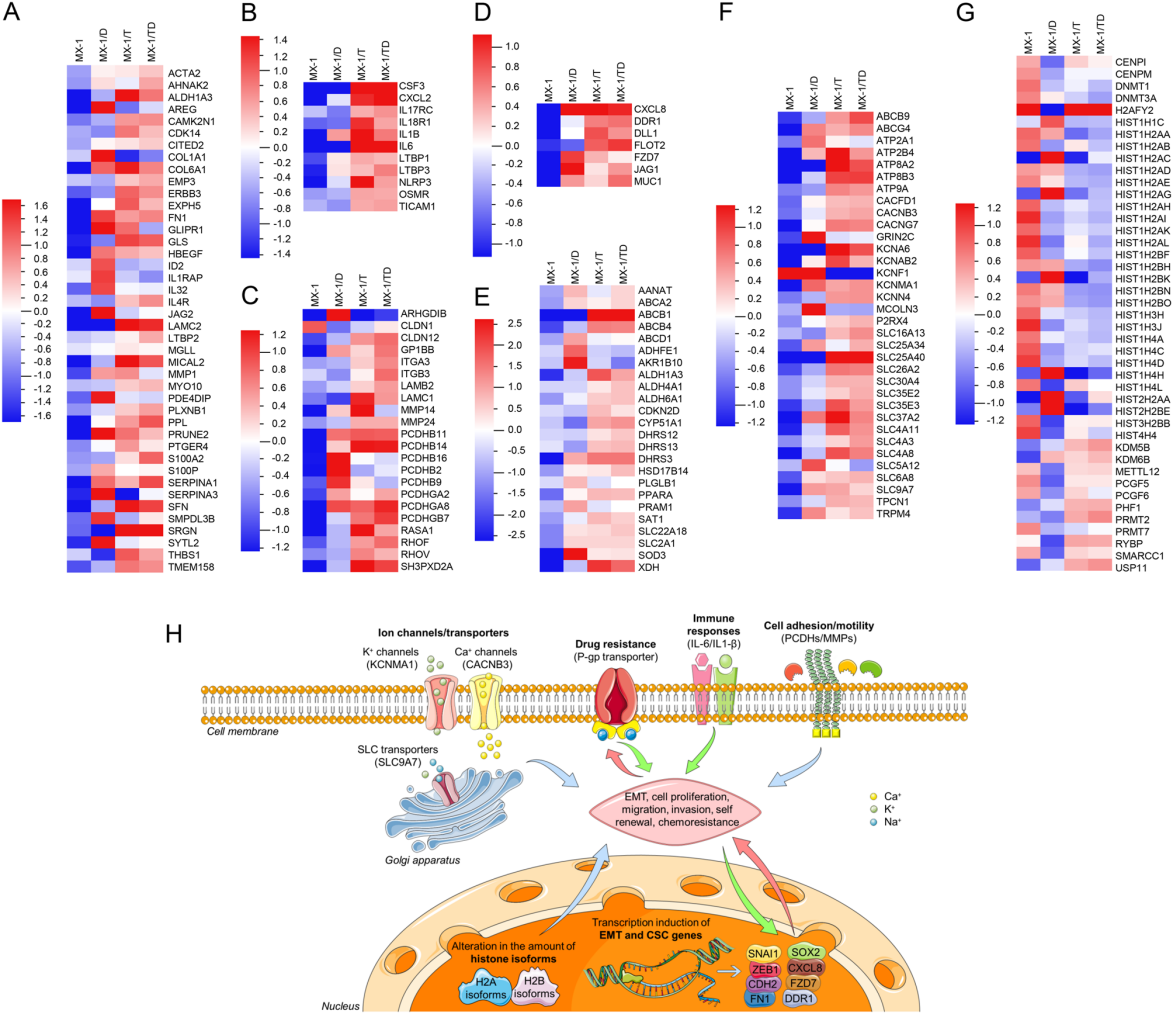
Cellular responses/pathways induced in chemoresistant MX-1 cell sublines. Heatmaps of differentially expressed genes (adjusted *p* < 0.05) from gene expression microarrays of chemoresistant MX-1 cell sublines: (**A**) epithelial–mesenchymal transition-related, (**B**) immune response-related, (**C**) cell adhesion and motility-related, (**D**) stem cells-related, (**E**) chemoresistance-related, (**F**) ion channels and (**G**) epigenetic regulators. The scale-bars indicate the normalized mRNA expression level, where the zero represents the mean expression level of the particular gene set. (**H**) The two hypothetical models of chemoresistance development were made according the human gene expression microarrays (Additional file 1, Table S1) and quantitative PCR results. The different coloring and directions of the arrows reflect the possibly different sequence of the activation of particular response pathway. In DOX-only exposed cells (red arrows) the deregulated expression of canonical EMT related genes and CSC markers was observed, what possibly caused cell migration and chemoresistance with an upregulated expression of drug metabolism-related enzymes (e.g. DOX metabolism-related *AKR1B10*) and later activation of *ABCB1* mRNA expression. In TPP^+^-exposed cells (green arrows) marked activation of *ABCB1* and immune response related genes were observed what supposedly caused cell migration, chemoresistance and possibly later activation of non-canonical EMT and CSC-related genes. The blue arrows indicate the pathways activated in both DOX-only and TPP^+^ treated cells. This figure was created using images from Servier Medical Art Commons Attribution 3.0 Unported License (http://smart.servier.com). Servier Medical Art by Servier is licensed under a Creative Commons Attribution 3.0 Unported License. *EMT* epithelial–mesenchymal transition, *CSC* cancer stem cell, *PCDHs* protocadherins, *MMPs* matrix metalloproteinases.

In order to understand the most enriched pathways and to identify deregulated gene signatures in chemoresistant cells compared to their parental counterparts, GSEA and IPA software were used. Analysis of deregulated genes revealed that despite the differences in gene expression patterns, almost identical response pathways were involved in the chemoresistance development of all cell sublines. Seven major pathways were deregulated during chemoresistance development: epithelial–mesenchymal transition, cancer stem cells, cell adhesion and motility, immune response, chemoresistance-related genes, various channels and transporters, as well as epigenetic regulators (Fig. 2A–H; Additional file 1, Table S1).

Among chemoresistance-related genes, the top upregulated genes were aldo–keto reductase *AKR1B10*, aldehyde dehydrogenase *ALDH1A3*, and xanthine dehydrogenase *XDH* (Additional file 1, Table S1). Marked up-regulation of various ion channels and transporters was determined in all chemoresistant cells, predominantly including calcium and potassium channels as well as SLC family transporters, while intense (FC > 6 × 10^3^) upregulation of *ABCB1* was observed in MX-1/T and MX-1/TD, but not in MX-1/D sublines.

The epithelial–mesenchymal transition pathway, as well as the cell adhesion and motility CSC pathway, were enriched in all chemoresistant sublines, but involved a wide spectrum of different genes (Fig. 2 and Additional file 1, Table S1). In contrast, chemokine encoded by *CXCL8* (FC ≥ 39), which is also considered as a CSC-mark, was upregulated in all chemoresistant sublines.

Some pathways were selectively activated as a response to DOX or TPP^+^. Immune response-related genes were predominantly upregulated in TPP^+^-exposed cell lines, with a > 200-fold increase in expression levels of interleukins *IL6* and *IL1B* in MX-1/T subline (Additional file 1, Table S1). In contrast, marked changes in expression and the spectrum of histones and epigenetic regulators encoding genes prevailed in all DOX treated cells (MX-1/D and MX-1/TD).

### Expression of EMT and cell stemness genes

**Figure 3:**
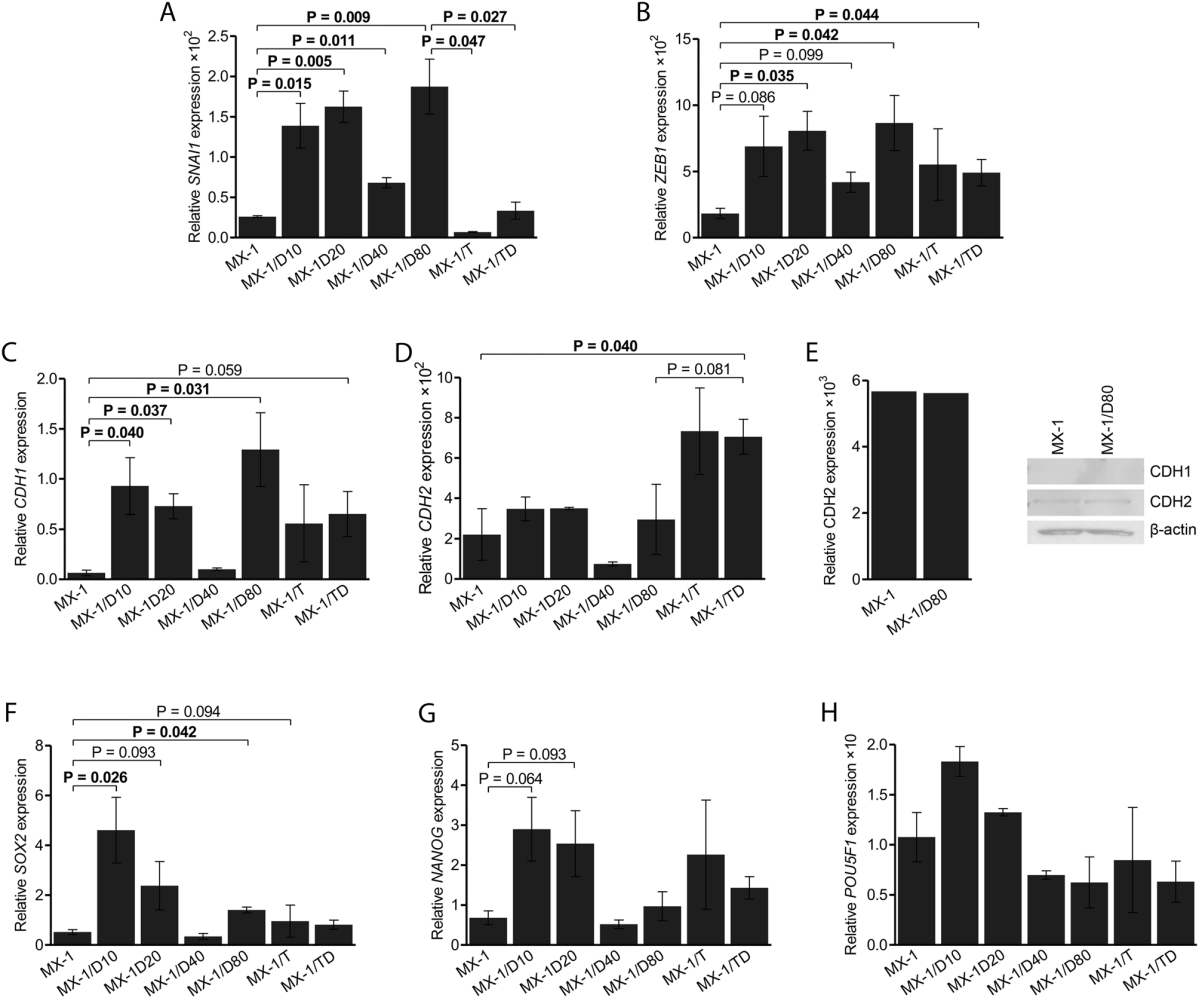
Differential gene expressions of the chemoresistant MX-1 cell sublines. (**A–D**) Results from quantitative PCR (qPCR) analysis showing the comparative mRNA expression of Epithelial–mesenchymal transition related genes in the parental MX-1 and chemoresistant cells, cultured with increasing concentrations (10 nM, 20 nM, 40 nM and 80 nM) of doxorubicin (MX-1/D), ABCB1 transporter activator TPP^+^ (MX-1/T) or both (MX-1/TD). (**E**) Western blot analysis of the protein expression of CDH1 and CDH2 in MX-1 and MX-1/D80 cells. (**F–H**) Relative expression level of cancer stem cells related genes. The graphs show the mean (± SEM) ratio of the indicated gene expression compared to endogenous control *HPRT1* expression. Indicated P values were calculated based on paired t-tests.

In an attempt to validate the involvement of the EMT process and to determine the sequence of events, the expression levels of key EMT-related (*CDH1*, *CDH2*, *SNAI1* and *ZEB1*) and cell stemness genes (*POU5F1*, *SOX2* and *NANOG*) were measured in MX-1/D sublines resistant to increasing concentrations of DOX, and compared to MX-1/T and untreated MX-1 cells (Fig. 3).

Upregulation of *SNAI1* and *ZEB1* were observed in MX-1/D cells, even at low concentrations of the drug, and reached 7.1-fold (*P* = 0.009) increase for *SNAI1* and 4.7-fold (*P* = 0.042) for *ZEB1* in MX-1/D80 subline (Fig. 3A,B). Increased mRNA level of *CDH1* was also observed in MX-1/D80 sublines, but was undetectable in protein level (FC = 21.4, *P* = 0.031; Fig. 3C,E; Additional file 1, Fig. 2). No significant changes in CDH2 expression were detected in mRNA or protein level (Fig. 3D,E). No significant alterations of the EMT gene expression were observed in MX-1/T as compared to untreated MX-1 cells, while the comparison to MX-1/D80 cells revealed significantly lower expression of *SNAI1* in MX-1/T, which was also characteristic of MX-1/TD cells. Differently from MX-1/T cells, MX-1/TD showed increased expression of *ZEB1* (FC = 2.6; *P* = 0.044; Fig. 3B) and *CDH2* (FC = 3.8; *P* = 0.040; Fig. 3D).

Upon selection for DOX resistance, an elevated expression of all evaluated stemness genes was observed (Fig. 3F–H) with the most evident upregulation of *SOX2* (*P* < 0.050) and *NANOG* (*P* = 0.064) in low concentrations of the drug. MX-1/T cells showed no expression changes of these genes.

### Hyperactivated expression of *ABCB1*

**Figure 4:**
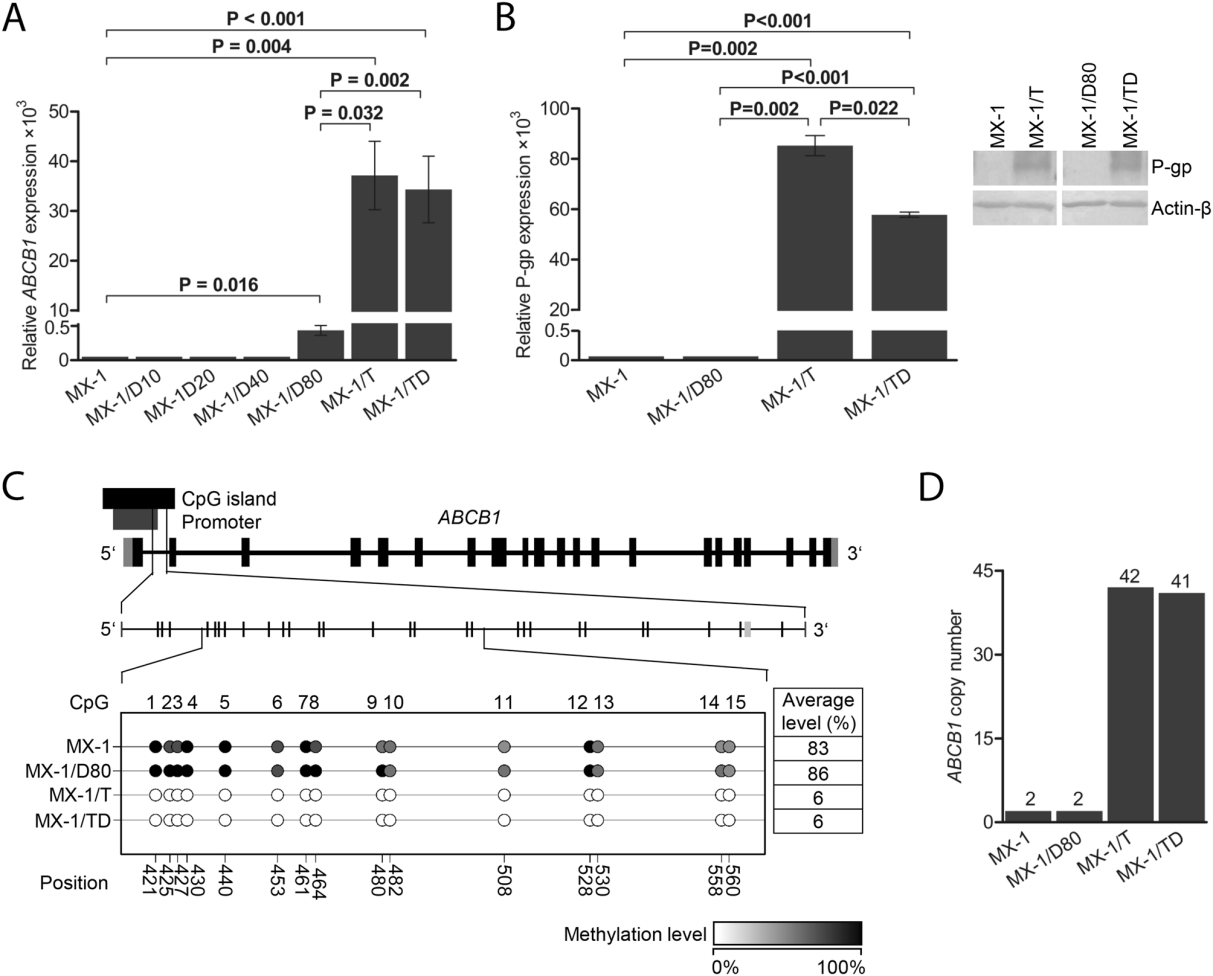
Upregulation of ABCB1 transporter in chemoresistant MX-1 cells. (**A**) mRNA levels of *ABCB1* in the MX-1 and chemoresistant cells, cultured with increasing concentrations (10 nM, 20 nM, 40 nM and 80 nM) of doxorubicin (MX-1/D10-80), ABCB1 transporter activator TPP^+^ (MX-1/T) or both (MX-1/TD) obtained by qPCR. The graph shows the mean (± SEM) ratio of the *ABCB1* expression compared to endogenous control *HPRT1* expression. (**B**) P-glycoprotein expression level in the parental MX-1 and chemoresistant MX-1/D80, MX-1/T and MX-1/TD cells obtained by Western blot analysis and the representative images of the experiment. The graphs show the mean (± SEM) ratio of the protein expression compared to actin β expression. *P* values were calculated based on paired t-tests. (**C**) Schematic figure detailing the CpG dinucleotides analysed by pyrosequencing in the downstream promoter of *ABCB1* gene and the methylation level of the selected CpGs in MX-1 and chemoresistant MX-1/D80, MX-1/T and MX-1/TD cells, depicted in the colours and expressed by percentage. (**D**) The copy number of *ABCB1* in non-treated MX-1 and chemoresistant MX-1/D80, MX-1/T and MX-1/TD cells evaluated by qPCR. The number of copies was calculated with Copy Caller Software v2.0 using *RNaseP* as an internal control.

For a more precise evaluation, *ABCB1* expression at mRNA and protein levels was evaluated in MX-1 sublines. Upon induction of DOX resistance, no significant alterations in *ABCB1* expression were observed at intermediate concentrations of DOX, and only MX-1/D80 cells showed marked upregulation of *ABCB1* mRNA (FC = 8.7 × 10^3^, *P* = 0.016; Fig. 4A). In MX-1/T cells, considerable upregulation of *ABCB1* was determined at both mRNA and protein levels (FC = 7.8 × 10^5^, *P* = 0.004 and FC = 8.5 × 10^4^, *P* = 0.002, respectively; Fig. 4A,B; Additional file 1, Fig. 3), compared to untreated cells. The similar *ABCB1* and P-gp expression pattern was observed in MX-1/TD cells as well (FC = 6.9 × 10^5^, *P* < 0.001 and FC = 5.7 × 10^4^, *P* < 0.001, respectively; Fig. 4A,B).

In the comparison with MX-1/D80 cells, *ABCB1* mRNA and protein level was significantly higher in MX-1/T (FC = 90.1, *P* = 0.032 and FC = 8.5 × 10^4^, *P* = 0.002, respectively) and MX-1/TD (FC = 79.4, *P* = 0.002, and FC = 5.7 × 10^4^, *P* < 0.001, respectively) cells (Fig. 4A,B). In contrast, only weak (FC = 1.6; *P* ≤ 0.040) upregulation of other ABC transporter ABCC1 was established in all MX-1 sublines (Additional file 1, Fig. 4).

### Genetic and epigenetic regulation mechanisms of *ABCB1*

To clarify the mechanism of this atypical ABCB1 production in MX-1/T and MX-1/TD cells, changes in the promoter DNA methylation status and gene copy number were analyzed. Pyrosequencing of 15 CpGs in the downstream promoter of the gene revealed a marked reduction of DNA methylation levels in MX-1/T and MX-1/TD sublines (from ≥ 83% down to 6%) as compared to MX-1/D80 or untreated cells (all P < 0.001; Fig. 4C). Furthermore, > 40 copies of *ABCB1* gene were detected in both MX-1/T and MX-1/TD sublines, while only two copies were present in untreated cells (Fig. 4D). In comparison, neither gene amplification nor promoter demethylation occurred in MX-1/D80 cells.

## Discussion

Drug resistance is the major obstacle to the successful treatment of cancer. Prolonged exposure to chemotherapeutic drugs causes cellular changes leading to the acquired ability of cancer cells to survive drug effect; however, in some instances, the resistance develops quite rapidly. Recent studies^19^ suggest that inherent features of cancer cells can accelerate chemoresistance development and these features exist even in chemotherapy-naive cancer cells. The inherent tissue-specific capacity to extrude xenobiotic compounds, including chemotherapeutic drugs, is markedly enhanced in cancer cells through various pathways that include ABC transporter hyperactivation. In our study, at least two possible scenarios of chemotherapeutic drug DOX-resistance were described in unique cell sublines with chemoresistance developed under pressure of DOX and the nongenotoxic activator of ABCB1 transporter—TPP^+^. The gradual activation of the canonical EMT pathway was shown during the development of DOX-induced resistance without significant changes in P-glycoprotein expression. Meanwhile, in the case of ABCB1-hyperexpression, observed in TPP^+^-exposed cells, EMT pathway was likely dispensable. Besides, the mechanism of this nongenotoxic xenobiotic pressure-induced overproduction of ABCB1, was quite unique and combined the two (epi)genetic phenomena, i.e. the increase in gene copy number and DNA hypomethylation in the promoter region.

DOX is an anthraquinone-type genotoxic chemotherapeutic drug commonly used for the treatment of a wide variety of cancers, including breast cancer^20,21^. The mechanism of DOX action consists of DNA intercalation followed by topoisomerase 2 inhibition and generation of free radicals^22,23^. Under the pressure of increasing concentration of the drug, the DOX-resistant MX-1 cell line was derived, showing a 38-fold higher resistance than the parental cell line. The process was accompanied by the evident morphology and gene expression changes specific to EMT. During the EMT, polarized epithelial cells of carcinoma undergo major phenotypic changes by acquiring mesenchymal morphology and CSC features^24,25^. Recent studies^26,27^ revealed that the chemotherapy-induced EMT is mainly responsible for the chemoresistance development, not only by the cancer dissemination as suggested previously^24,28^, but increasing expression of ABC transporters has been related to this process as well, owing to the activity of the EMT-inducing transcription factors (TFs) on the promoters of ABC transporters^2,29,30^. In our study, the development of DOX-resistance in the MX-1 cell line, nicely conformed to this scenario. First, the key EMT TFs *SNAI1* and *ZEB1* together with stem cell markers *SOX2* and *NANOG* were activated, and then this was followed by increased *ABCB1* expression*,* however, without significant changes at the protein level. Moreover, this process involved multiple changes in the EMT, cell stemness, and chemoresistance pathways as observed in genome-wide gene expression analysis. However, the process of chemoresistance development can be led not only by the EMT.

There are reports showing the ability of ABCB1 to influence the EMT, and inhibition or decreased expression of *ABCB1* can lead to its suppression^31,32^. In our study, the treatment of MX-1 cells with a typical substrate for ABC transporters—TPP^+^—resulted not only in significant upregulation of the *ABCB1* gene, but also more than a 70-fold increase in resistance to DOX- and induced EMT-specific morphology changes. TPP^+^ is a non-genotoxic compound that tends to accumulate in mitochondria^33^. Attempts are made to use this lipophilic cation as a tracker moiety to deliver antioxidants to mitochondria for the prevention of senescence and age-related diseases, and also for targeted delivery of drugs in cancer chemotherapy^33–35^. In our study, DOX-resistance activated by this xenobiotic was accompanied by the most abundant gene expression changes and dysregulation of various cellular pathways, but the strongest effect of this compound was observed on *ABCB1* expression.

ABCB1 is considered as the key efflux pump, extruding various drugs and xenobiotics from the cells^36^ and the contribution of this protein to chemoresistance was discovered years ago^37^. Our study suggests that treatment of MX-1 cells with increasing concentrations of DOX can eventually activate expression of *ABCB1*, which is in agreement with the previous study^29^. However, the cell pretreatment with nongenotoxic ABC transporter substrate TPP^+^ can considerably enhance this process. Moreover, TPP^+^-induced overexpression of ABCB1 is driven by both genetic and epigenetic phenomena. Previous studies have shown the positive correlation between *ABCB1* promoter hypomethylation^13^ or the gene amplification^14^ and its expression level. In our study, *ABCB1* hyperexpressing cells were characterized by both gene amplification and DNA demethylation in the downstream promoter; the latter mechanism potentially is decisive, because tremendous > 7 × 10^5^-fold expressional upregulation of *ABCB1*, observed in this study, cannot be explained only by 21-fold increased gene amplification. To the contrary, no such genetic or epigenetic alterations were found in DOX-only treated cells, where *ABCB1* expression was possibly enhanced by another possible mechanism involving the EMT-specific TFs.

## Conclusions

The results of our study suggest two possible pathways towards chemoresistance: through activation of the key EMT TFs without the need of ABCB1 activation and through ABCB1 hyperactivation by both, genetic and epigenetic mechanisms. According to our observation, the combination of demethylation of the *ABCB1* downstream promoter and gene amplification plays the crucial role for acquisition of stronger chemoresistance. This study showed the possibility, that controlling at least one of these mechanisms may lead to a way to overcome the chemoresistance. Besides, the data of our study warn about the possibility of accelerated development of chemoresistance by exposure to nongenotoxic ABCB1 inducers. Further, in vivo studies could assist to elucidate the exact response pathways activated by such ABCB1 inducers.

## Materials and methods

### Cell cultivation and establishment of resistant sublines

The MX-1 cell line was maintained as an attached monolayer culture in RPMI 1640 medium, supplemented with 10% (v/v) heat-inactivated fetal bovine serum (FBS), 100 U/mL and 100 μg/mL penicillin–streptomycin (all from Biochrom AG, Germany). The cells were grown in cell culture bottles (growth surface 75 cm^2^) with filter screw caps (Carl Roth, Germany) at 37 °C in an atmosphere with 90% humidity and 5% CO_2._ For the investigation of the mechanisms of chemoresistance, cell models—DOX-exposed and TPP^+^-exposed MX-1 cells—were established and named MX-1/D and MX-1/T, respectively. Drug resistance was derived by a stepwise selection method as described previously^38^. Briefly, initially DOX-sensitive MX-1 cells were incubated with 10 nM DOX (Teva, UK), and doubling of the drug concentration was performed until the final concentration of 80 nM DOX was applied. Ultimately, four MX-1 cell sublines resistant to increasing concentrations of DOX were established and named MX-1/D10, MX-1/D20, MX-1/D40, and MX-1/D80, accordingly. These MX-1/D cell sublines were cultured in the indicated concentrations to maintain the resistance. For generation of ABCB1-active MX-1 cells, increasing concentrations (from 2 to 128 μM) of TPP^+^ (chloride salt, Fluka, St. Gallen, Switzerland), as a model substrate of ABC transporters^18^, were used and cells resistant up to 128 μM of TPP^+^ were generated (MX-1/T). Finally, ABCB1-active cells were exposed to high concentrations (up to 1280 nM) of DOX for a week in order to generate MX-1/TD cell sublines. The morphological changes in all derived chemoresistant MX-1 cell sublines were observed by an inverted fluorescence microscope Motic AE31 (Xiamen, China). The images were captured at a 200-fold magnification.

### Cell viability measurements

Cell chemoresistance was determined as cell survival in the exposure to various DOX concentrations by standard colorimetric MTT assay as previously described^39^. Cells were seeded into 96-well plates with each well receiving a volume of 200 μL at a density of 10^4^ cells/well. Then DOX at a final concentration of 0.05–51.2 µM, paclitaxel (Pharmachemie B.V., Netherlands) and cisplatin (Accord healthcare, Estonia) at final concentrations of 0.065–64.0 µM was added to the test cells and no drugs was added to the control. Cells were cultured in plates at 37 °C under a humidified atmosphere with 5% CO_2_ for 72 h. MTT was dissolved in PBS at 5 mg/mL and then medium with DOX was exchanged by 20 μL of this solution. The cells were incubated at 37 °C for another 1 h, after which cells were washed 3 times with PBS, and 50 μL of 2-propanol was added to each well. The absorbance at 585 nm was measured using a Tecan GeniosPro plate reader with 612 nm as a reference wavelength. The absorbance of the background was determined using 2-propanol but no cells. The results are presented as the percentage viability of cells exposed to DOX relative to non-exposed cells.

### RNA and DNA extraction

For the isolation of nucleic acids, chemoresistant MX-1/D, MX-1/TD cells were grown in DOX supplemented medium, while MX-1/T cells with TPP^+^. At least 1 × 10^6^ cells were used for genomic DNA extraction using NucleoSpin Tissue Kit according to the manufacturer’s protocol (Macherey–Nagel, Germany). The total RNA was extracted from about 2 × 10^6^ cells with mirVana Kit according to the manufacturer’s protocol (Ambion, Thermo Fisher Scientific, Foster City, CA, USA). The RNA integrity number (RIN) was ≥ 9.4, as evaluated with 2100 Bioanalyzer system using RNA 6000 Nano Kit (Agilent Technologies, Santa Clara, CA, USA).

### Global gene expression profiling

Microarray hybridization was performed according to the manufacturer’s protocol for One-Color Microarray-Based Gene Expression Analysis v6.5 (Agilent Technologies) as described previously^40^. Briefly, 200 ng of total RNA was processed with a Low Input Quick Amp Labeling Kit, One Color and using a RNA Spike-In Kit, One Color (Agilent Technologies). Samples were hybridized onto Human Gene Expression (v2) 8 × 60 K microarrays (design ID 039494; Agilent Technologies) for 17 h at 65 °C. Microarrays were scanned using a SureScan microarray scanner, and the images were analyzed with Feature Extraction software v10.7 (Agilent Technologies). Data have been submitted to Gene Expression Omnibus repository, record number GSE163986. GeneSpring software v12.6 (Agilent Technologies) was used for data pre-processing from two separate experiments. Individual probe signals marked as saturated, non-uniform, or outlier were marked NA. Probes having an NA within at least one sample were removed from the analysis. The dataset of each sample was log2-transformed and 75-percentile normalized without baseline transformation. Probe annotations were extracted from eArray platform [https://earray.chem.agilent.com] according to the corresponding microarray design identifier. For the comparison of two groups, the fold change (FC) value was estimated and t-test was applied. Pathway analysis was performed using the Gene Set Enrichment Analysis software (GSEA) developed by the Broad Institute^41^. GSEA provides a software platform to analyze gene expression data through pairwise comparison of the dataset of interest with known biological processes and pathways. Our dataset was compared against the following reference genesets: hallmark epithelial mesenchymal transition, immune and inflammatory response, xenobiotic metabolism, KEGG ABC transporters, drug metabolism—other enzymes, cell adhesion molecules cams, ECM receptor interactions, GO regulation of cell differentiation, transporter activity, cellular component movement, and chromosome organisation. Data was also analyzed with the Ingenuity Pathway Analysis (IPA, QIAGEN Inc., https://www.qiagenbioinformatics.com/products/ingenuity-pathway-analysis). IPA software examines functional relationships within an input list of genes, and identifies the pathways from the IPA library of canonical pathways that were most significantly associated with the dataset.

### Target gene expression profiling by RT-qPCR

For reverse transcription (RT), Maxima First Strand cDNA Synthesis Kit for RT-qPCR was used according to the manufacturer’s protocol (Thermo Scientific, Thermo Fisher Scientific). Aliquots containing 1 μg of total RNA were used from each sample. Expression levels of selected genes—*ABCB1*, *ABCC1*, *POU5F1*, *NANOG*, *SOX2*, *CDH1*, *CDH2*, *SNAI1*, *ZEB1*, and endogenous control *HPRT1*– were evaluated in all cell sublines using TaqMan Gene Expression Assays Hs_00184500_m1, Hs_01561502_m1, Hs_00999632_g1, Hs_04260366_g1, Hs_04260357_g1, Hs01023895_m1, Hs00983056_m1, Hs00195591_m1, Hs00232783_m1, and Hs02800695_m1, respectively (all from Applied Biosystems, Thermo Fisher Scientific). Quantitative PCR (qPCR) was performed in duplicates on ViiA 7 Real Time PCR System (Applied Biosystems, Thermo Fisher Scientific). Reaction volume of 20 μL consisted of 1 × TaqMan Universal Master Mix II, no UNG, 2 × TaqMan Gene Expression Assay (both from Applied Biosystems, Thermo Fisher Scientific) and 2 μL of cDNA sample. Thermocycling parameters were 95 °C for 10 min, followed by 40 cycles of 95 °C for 15 s and 60 °C for 1 min. Two technical replicates of the experiment starting from the RT step were performed. For the analysis of relative changes in gene expression, raw cycle of quantification (Cq) values were normalized to *HPRT1* and converted to linear scale.

### DNA methylation analysis by pyrosequencing

Four hundred ng of isolated DNA was modified with bisulfite using the EZ DNA Methylation Kit (Zymo Research, USA) and standard protocol, except that the initial incubation of samples was performed at 42 °C for 15 min. DNA methylation intensity in *ABCB1* gene was analyzed by pyrosequencing, using primers designed with PyroMark Assay Design Software (version 2.0.1.15, Qiagen, Valencia, CA, USA). The target sequence contained 15 CpGs in total. The PCR mixture (25 μL) consisted of 1 × PyroMark PCR Master Mix (Qiagen), 0.28 μM of each primer (Forward: biotin-GTGGGTGGGAGGAAGTAT and Revers: ATCCCCTTCAAAATCCATTCC) and 3 μL bisulfite modified DNA. Prior to pyrosequencing, PCR products were analyzed on 3% agarose gel with ethidium bromide staining. Single-stranded amplicons from 10 μL of PCR products were isolated using Streptavidin Sepharose High Performance mix (GE Healthcare Bio-Sciences AB, Uppsala, Sweden). Pyrosequencing was performed with the PyroMark Q24 system (Qiagen) using PyroMark Q24 Gold reagents (Qiagen) and 0.91 μL sequencing primers (5′-CTAACAACCCCTTCTAAACTTTACC-3′ and 5′-ATACCCCAACTACTCTAA-3′). Data was analyzed using the PyroMark Q24 Software (version 2.0.6, Qiagen) which calculates the methylation intensity as a C/T ratio at each CpG position.

### *ABCB1* copy number quantification

To assess the *ABCB1* gene copy number, TaqMan Copy Number Assay (Hs04939312_cn) was used. For the internal control RNaseP TaqMan Copy Number Reference Assay (assay ID 4403326) was selected. The assays were performed in duplicates using StepOne Plus PCR instrument (Applied Biosystems, Thermo Fisher Scientific). Reaction mix (20 μL) consisted of 1 × TaqMan Universal Master Mix II, no UNG, 2 × TaqMan Copy Number Assay, 2 × TaqMan Copy Number Reference Assay (all from Applied Biosystems, Thermo Fisher Scientific) and 20 ng of the DNA sample. Thermocycling parameters consisted of 95 °C for 10 min, followed by 40 cycles of 95 °C for 15 s and 60 °C for 1 min. Relative quantification analysis was done to estimate the *ABCB1* copy number for each sample by using the Copy Caller Software v2.0 (Applied Biosystems).

### Western blot analysis

Western blot analysis was performed as previously described^42^. Briefly, chemoresistant MX-1/D and MX-1/TD cells were grown in a DOX-supplemented medium, while MX-1/T cells were grown with TPP^+^. Total proteins were isolated from cell lysates in RIPA buffer which contained 50 mM Tris–HCl (pH 7.5), 150 mM NaCl, 1% IGEPAL CA-630 (Sigma-Aldrich; Merck Millipore, Darmstadt, Germany), 0.5% sodium deoxycholate and 0.1% sodium dodecyl sulfate (SDS), protease inhibitor cocktail (Sigma-Aldrich; Merck Millipore). Cells were homogenized using an ultrasonic sonicator (500-W Ultrasonic Processor; Cole-Parmer Instrument Co. Ltd., London, UK). Protein samples were separated onto 7.5% SDS–polyacrylamide gel for the detection of P-gp and 8% for the detection of CDH1 and CDH2 and transferred to nitrocellulose membranes (Sigma-Aldrich, Steinheim, Germany). Proteins were labelled with anti-P-gp, anti-CDH1, anti-CDH2 or anti-β-actin (Invitrogen, Thermo Fisher Scientific, Vilnius, Lithuania) antibodies in blocking solution (5% non-fat milk in phosphate-buffered saline, PBS). Immunoreactive bands were detected by anti-mouse HRP-conjugated secondary antibody (Invitrogen, Thermo Fisher Scientific, Vilnius, Lithuania). Immunocomplexes were visualized using liquid 3,3′,5,5′-tetramethylbenzidine substrate (Sigma-Aldrich; Merck Millipore) and documented using the CanoScan 5600F (Canon, Thailand) scanner. The values of P-gp, CDH1 and CDH2 signals were calculated using the image analysis program ImageJ version 1.47 (National Institutes of Health, Bethesda, USA).

### Statistical analysis

Statistical analysis was performed using STATISTICA v8.0 (StatSoft, Tulsa, OK, USA) and GraphPad Prism software v0.0 (GraphPad Software, La Jolla, CA, USA). Data are statistically presented as the mean (± Standard Error of the Mean (SEM)) for at least two separate experiments. Comparisons between groups were analyzed via t-test^29^. Probability values of *P* < 0.050 were considered statistically significant.

## Supplementary Information

Supplementary Information.

## Data Availability

The datasets supporting the conclusions of this article are included within the article and its supplementary information.

## Abbreviations

CSC: Cancer stem cells
DOX: Doxorubicin
EMT: Epithelial–mesenchymal transition
TPP^+^: Tetraphenylphosphonium cation
