# Supplementary material for: Nongenotoxic ABCB1 activator tetraphenylphosphonium can contribute to doxorubicin resistance in MX-1 breast cancer cell line
Source: Sci Rep. 2021 Mar 22;11:6556. doi: 10.1038/s41598-021-86120-6 (PMC7985213; doi:10.1038/s41598-021-86120-6)
Supplement: Supplementary file 1 — Supplementary Information. [file 41598_2021_86120_MOESM1_ESM.docx]

**Nongenotoxic ABCB1 activator tetraphenylphosphonium can contribute to doxorubicin resistance in MX-1 breast cancer cell line**

Raimonda Kubiliute^1,2,3^, Indre Januskeviciene^2^, Ruta Urbanaviciute^2^, Kristina Daniunaite^1,3^, Monika Drobniene^3^, Valerijus Ostapenko^3^ Rimantas Daugelavicius^2^, Sonata Jarmalaite^1,3^

^1^Institute of Biosciences, Life Sciences Center, Vilnius University, LT‑10257 Vilnius, Lithuania.

^2^Department of Biochemistry, Faculty of Natural Sciences, Vytautas Magnus University, LT-44404 Kaunas, Lithuania.

^3^National Cancer Institute, LT‑08406 Vilnius, Lithuania.


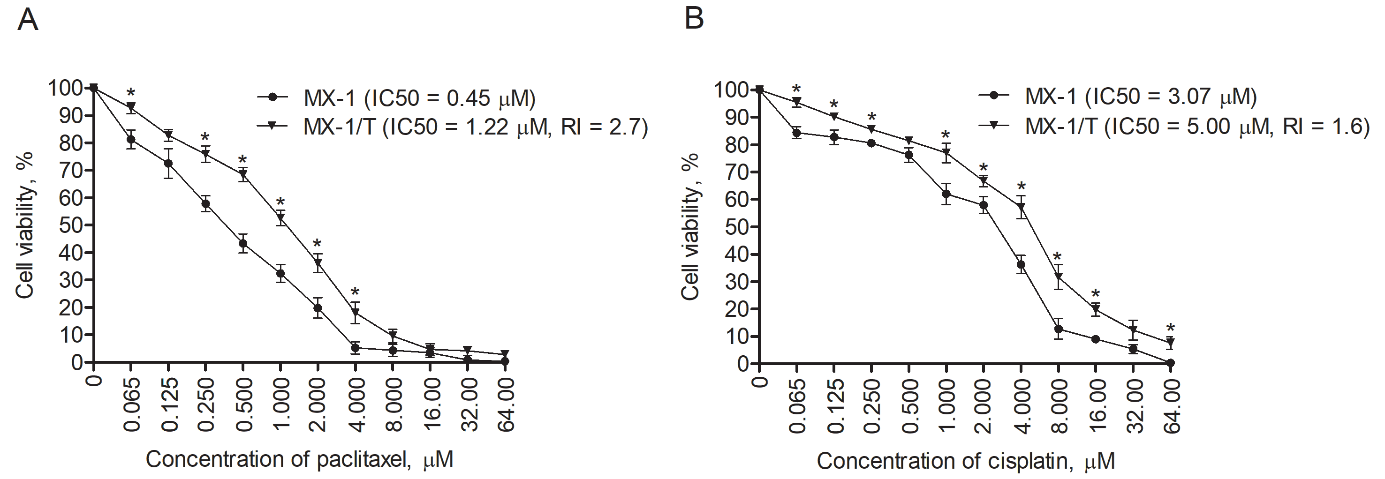


**Supplementary Figure1.** **Multidrug resistance of ABCB1-hyperexpressing MX-1/T cells**. Cytotoxicity of paclitaxel (A) and cisplatin (B) measurements with the MTT assay in the parental MX-1 cells and chemoresistant MX-1/T (cultured with ABCB1 transporter activator TPP+ (128 nM)) subline. Resistance index was calculated IC50 values of chemoresistant cells dividing by IC50 of parental MX-1 cells. The asterisks indicate statistically significant (P < 0.05) differences between wild-type and chemoresistant cells. RI - resistance index.

**
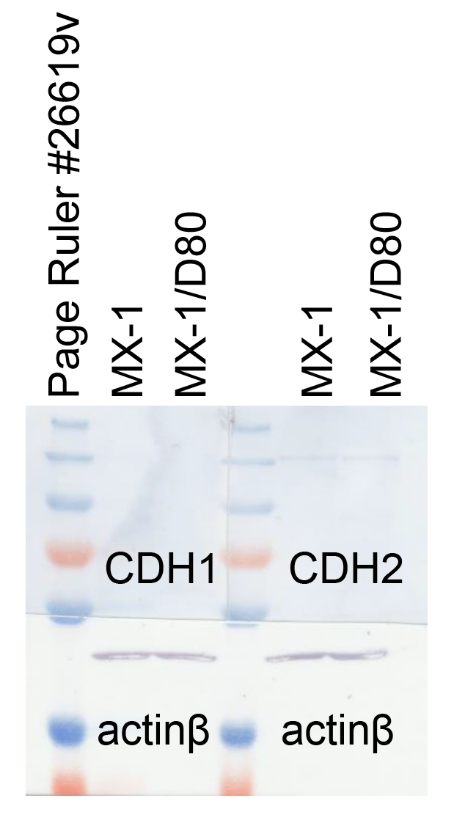
**

**Supplementary Figure 2.** **Images of Western blot analysis of CDH1 and CDH2 in MX-1 cell sublines.** CDH1, CDH2 and actinβ expression levels in the parental MX-1 and chemoresistant MX-1/D80 cells obtained by Western blot analysis.


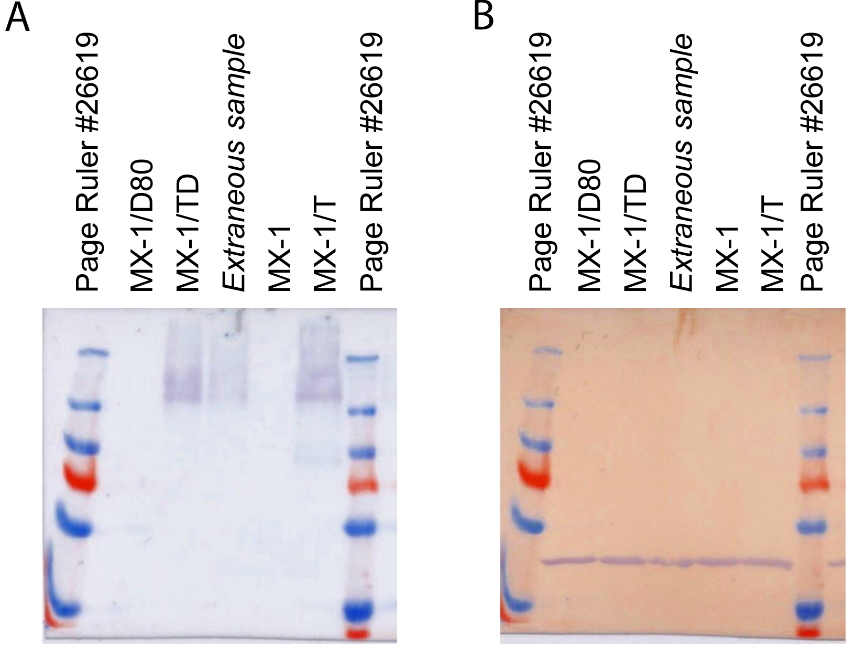


**Supplementary Figure 3.** **Images of Western blot analysis of Pgp in MX-1 cell sublines.** P-glycoprotein (A) and actinβ (B) expression levels in the parental MX-1 and chemoresistant MX-1/D80, MX-1/T and MX-1/TD sublines obtained by Western blot analysis.


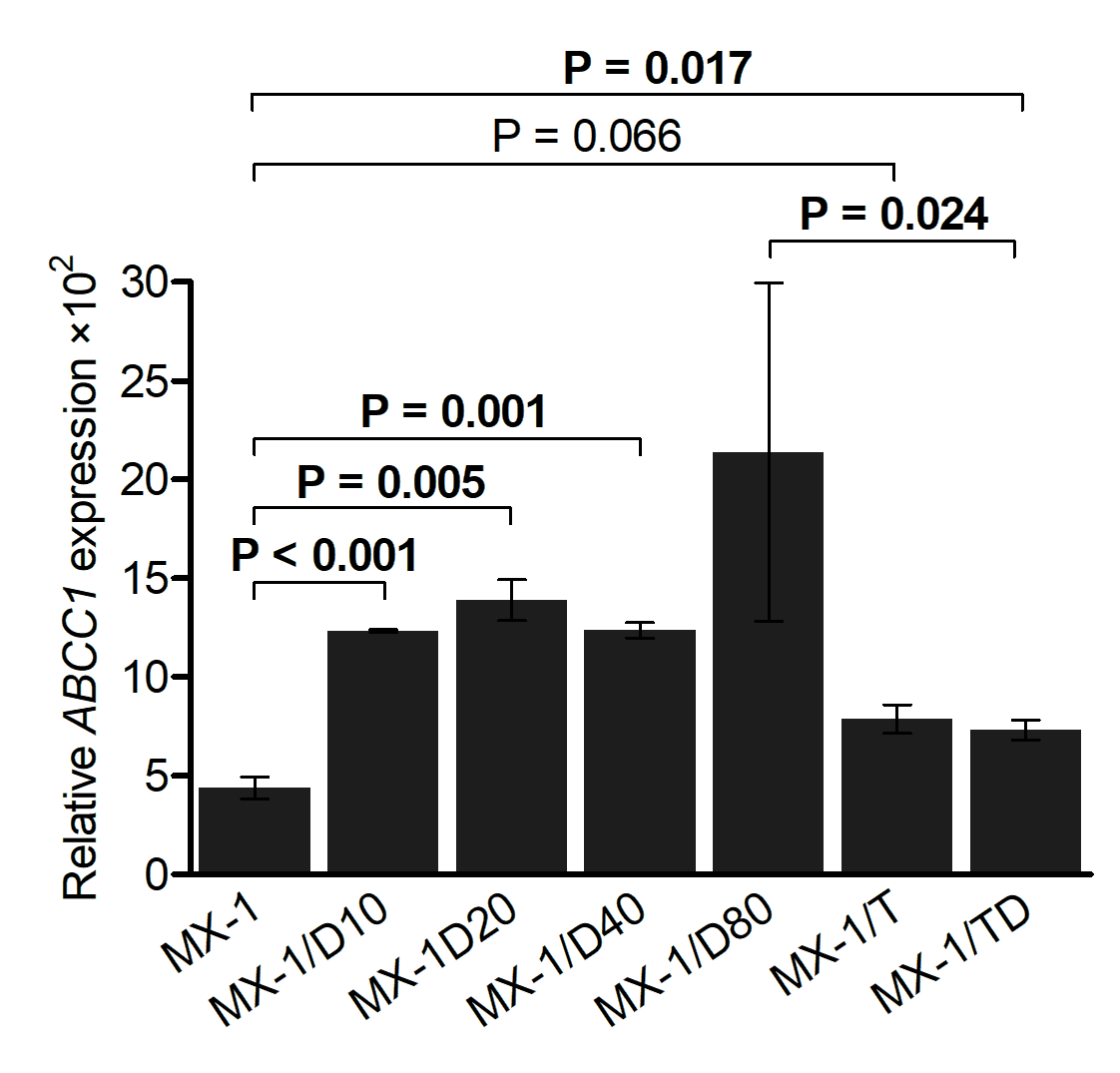


**Supplementary Figure 4. *ABCC1* exprssion in MX-1 cell sublines.** mRNA levels of *ABCB1* in the MX-1 and chemoresistant cells, cultured with increasing concentrations (10 nM, 20 nM, 40 nM and 80 nM) of doxorubicin (MX-1/D10-80), ABCB1 transporter activator TPP^+^ (MX-1/T) or both (MX-1/TD) obtained by qPCR. The graph show the mean (± SEM) ratio of the *ABCC1* expression compared to endogenous control *HPRT1* expression. P values were calculated based on paired t-tests.

**Supplementary Table S1.** Differentially expressed EMT, chemoresistance, cancer stem cells (CSC), immune response, cell adhesion and motility related genes as well as epigenetic regulators, various channels and transporters coding genes in chemoresistant MX-1/D (cultured with 80 nM of doxorubicin), MX-1/T (cultured with ABCB1 transporter activator TPP+ (128 nM)) and MX-1/TD (MX-1/T cells cultured in media supplemented with up to 1280 nM of doxorubicin for a week) cells compared to parental MX-1 cells. The results were obtained using Human Gene Expression (v2) 8×60K microarrays (design ID 039494) and selected manually, accordin to the result, obtained by Gene Set Enrichment Analysis, Ingenuity Pathway Analysis softwares and previously publishd studies. Indicated P values were calculated based on paired t-tests and only significant values are provided. FC - absolute fold change level.

|  | **MX-1/D vs. MX-1** | | | **MX-1/T vs. MX-1** | | | **MX-1/TD vs. MX-1** | | |
| --- | --- | --- | --- | --- | --- | --- | --- | --- | --- |
|  | FC | p-value | Regulation | FC | p-value | Regulation | FC | p-value | Regulation |
| **EMT-related genes** | | | | | | | | | |
| ABLIM1 | 2,1 | 0,043 | down | 3,0 | 0,042 | down | 2,5 | 0,031 | down |
| ACTA2 | - | - | - | - | - | - | 2,1 | 0,009 | up |
| AHNAK2 | 2,3 | 0,010 | up | - | - | - | 2,6 | 0,019 | up |
| ALDH1A3 | - | - |  | 18,1 | 0,000 | up | 9,5 | 0,016 | up |
| ANK3 | 3.3 | 0,010 | down | 2,6 | 0,029 | down | 2.6 | 0,011 | down |
| AREG | - | - | - | 2,0 | 0,002 | up | 3,4 | 0,046 | up |
| C10orf10 | - | - | - | 23,2 | 0,047 | up | 29,7 | 0,002 | up |
| C9orf3 | - | - | - | 4,1 | 0,044 | down | 4,0 | 0,010 | down |
| CALD1 | 5,4 | 0,037 | up | 6,4 | 0,033 | down | - | - | - |
| CAMK2N1 | - | - | - | - | - | - | 3,5 | 0,032 | up |
| CDK14 | - | - | - | 3,0 | 0,009 | up | - | - | - |
| CITED2 | - | - | - | 2,0 | 0,019 | up | - | - | - |
| COBLL1 | - | - | - | 5,3 | 0,032 | down | 3,6 | 0,032 | down |
| COL1A1 | 9,1 | 0,041 | up | - | - | - | - | - | - |
| COL6A1 | - | - | - | 36,6 | 0,017 | up | 22,4 | 0,037 | up |
| CXCL2 | - | - | - | - | - | - | 22,8 | 0,034 | up |
| DACT3 | 11,0 | 0,050 | up | - | - | - | - | - | - |
| DDR1 | - | - | - | 3,5 | 0,033 | up | 3,6 | 0,008 | up |
| DLC1 | 3,9 | 0,031 | up | - | - | - | - | - | - |
| DSC2 | - | - | - | 3,0 | 0,027 | up | 3,8 | 0,004 | up |
| EMP3 | - | - | - | - | - | - | 2,0 | 0,003 | up |
| ENPP1 | - | - | - | 10,1 | 0,034 | down | - | - | - |
|  | **MX-1/D vs. MX-1** | | | **MX-1/T vs. MX-1** | | | **MX-1/TD vs. MX-1** | | |
|  | FC | p-value | Regulation | FC | p-value | Regulation | FC | p-value | Regulation |
| **EMT-related genes** | | | | | | | | | |
| ERBB3 | - | - | - | - | - | - | 4,0 | 0,040 | up |
| ETV1 | 2,2 | 0,016 | down | - | - | - | - | - | - |
| EXPH5 | - | - | - | 7,1 | 0,016 | up | - | - | - |
| FBLN1 | - | - | - | - | - | - | 2,1 | 0,005 | down |
| FGF2 | - | - | - | - | - | - | 3,2 | 0,034 | down |
| FN1 | - | - | - | 7,0 | 0,004 | up | 14,2 | 0,036 | up |
| FZD7 | - | - | - | 3,3 | 0,002 | up | 2,8 | 0,034 | up |
| GEMIN2 | 2,1 | 0,022 | down | - | - | - | - | - | - |
| GLIPR1 | 61,4 | 0,027 | up | - | - | - | - | - | - |
| GLS | - | - | - | - | - | - | 6,6 | 0,034 | up |
| HBEGF | - | - | - | - | - | - | 5,4 | 0,033 | up |
| HTRA1 | - | - | - | 2,8 | 0,039 | up | 4,4 | 0,001 | up |
| ID2 | 2,9 | 0,030 | up | - | - | - | - | - | - |
| IFI44L | - | - | - | 15,2 | 0,016 | down | - | - | - |
| IL1B | - | - | - | 260,7 | 0,006 | up | 46,3 | 0,042 | up |
| IL1RAP | 2,9 | 0,046 | up | - | - | - | - | - | - |
| IL32 | 5,2 | 0,010 | up | - | - | - | - | - | - |
| IL4R | - | - | - | - | - | - | 2,4 | 0,020 | up |
| INPP4B | - | - | - | 7,6 | 0,050 | down | - | - | - |
| ITGB4 | - | - | - | - | - | - | 6,4 | 0,033 | up |
| JAG1 | - | - | - | - | - | - | 6,5 | 0,006 | up |
| JAG2 | 23,6 | 0,016 | up | - | - | - | 4,2 | 0,019 | up |
| LAMA3 | 21,9 | 0,018 | up | 134,5 | 0,005 | up | 121,4 | 0,000 | up |
| LAMC2 | - | - | - | 114,8 | 0,001 | up | - | - | - |
| LSR | 5,3 | 0,013 | down | - | - | - | - | - | - |
| LSS | 4,6 | 0,015 | down | - | - | - | - | - | - |
| LTBP1 | - | - | - | - | - | - | 2,9 | 0,023 | up |
| LTBP2 | - | - | - | - | - | - | 2,0 | 0,022 | up |
| MALL | - | - | - | 4,4 | 0,006 | down | 2,5 | 0,008 | down |
|  | **MX-1/D vs. MX-1** | | | **MX-1/T vs. MX-1** | | | **MX-1/TD vs. MX-1** | | |
|  | FC | p-value | Regulation | FC | p-value | Regulation | FC | p-value | Regulation |
| **EMT-related genes** | | | | | | | | | |
| MAN1A1 | - | - | - | 4,0 | 0,009 | down | - | - | - |
| MBP | - | - | - | 3,1 | 0,009 | up | - | - | - |
| MGLL | - | - | - | 33,1 | 0,027 | up | - | - | - |
| MICAL2 | - | - | - | - | - | - | 2,4 | 0,021 | up |
| MMP1 | 4,1 | 0,001 | up | 4,4 | 0,005 | up | - | - | - |
| MMP19 | - | - | - | - | - | - | 2,4 | 0,036 | down |
| MTSS1 | - | - | - | 6,3 | 0,032 | up | - | - | - |
| MTUS1 | - | - | - | 9,5 | 0,038 | down | - | - | - |
| MYO10 | - | - | - | - | - | - | 2,0 | 0,047 | up |
| NDRG1 | - | - | - | 7,2 | 0,038 | up | 5,3 | 0,009 | up |
| NRG1 | - | - | - | 2,0 | 0,010 | down | - | - | - |
| NT5E | 2,3 | 0,023 | down | - | - | - | - | - | - |
| PCDH7 | - | - | - | 2,7 | 0,002 | down | 2,6 | 0,006 | down |
| PDE4DIP | 6,5 | 0,000 | up | - | - | - | 2,1 | 0,031 | up |
| PLA2G4A | - | - | - | 39,1 | 0,035 | down | - | - | - |
| PLS1 | - | - | - | 2,5 | 0,050 | down | - | - | - |
| PLXNB1 | - | - | - | - | - | - | 2,8 | 0,037 | up |
| POLR3G | 2,6 | 0,009 | down | - | - | - | - | - | - |
| PPL | - | - | - | - | - | - | 8,9 | 0,010 | up |
| PRSS23 | - | - | - | 4,9 | 0,021 | down | 3,9 | 0,008 | down |
| PRUNE2 | - | - | - | - | - | - | 12,0 | 0,008 | up |
| PTGER4 | - | - | - | 3,0 | 0,013 | up | - | - | - |
| S100A2 | - | - | - | - | - | - | 3,6 | 0,020 | up |
| S100P | 2,4 | 0,045 | up | - | - | - | - | - | - |
| SERPINA1 | - | - | - | 10,7 | 0,001 | up | 17,5 | 0,001 | up |
| SERPINA3 | 30,7 | 0,041 | up | - | - | - | - | - | - |
| SFN | 3,6 | 0,037 | up | 17,0 | 0,027 | up | 13,8 | 0,049 | up |
| SLC39A8 | - | - | - | 125,2 | 0,002 | down | 60,1 | 0,000 | down |
| SMPDL3B | 5,6 | 0,006 | up | - | - | - | - | - | - |
|  | **MX-1/D vs. MX-1** | | | **MX-1/T vs. MX-1** | | | **MX-1/TD vs. MX-1** | | |
|  | FC | p-value | Regulation | FC | p-value | Regulation | FC | p-value | Regulation |
| **EMT-related genes** | | | | | | | | | |
| SPP1 | - | - | - | 704,0 | 0,014 | down | - | - | - |
| SRGN | 20,4 | 0,022 | down | 21,7 | 0,011 | up | 18,8 | 0,000 | up |
| SYTL2 | 8,9 | 0,048 | up | - | - | - | - | - | - |
| THBS1 | - | - | - | 2,8 | 0,039 | up | - | - | - |
| TLR3 | - | - | - | 2,6 | 0,011 | down | - | - | - |
| TMEM158 | - | - | - | 4,7 | 0,026 | up | 4,1 | 0,004 | up |
| TPM1 | - | - | - | 2,1 | 0,032 | down | - | - | - |
| TSPAN1 | - | - | - | - | - | - | 5,6 | 0,005 | up |
| VCAN | - | - | - | - | - | - | 12,7 | 0,032 | down |
| WNT5A | - | - | - | - | - | - | 2,3 | 0,045 | down |
| **Chemoresistance-related genes** | | | | | | | | | |
| AANAT | 3,4 | 0,011 | up | - | - | - | - | - | - |
| ABCA2 | 2,9 | 0,034 | up | 2,4 | 0,014 | up | 2,9 | 0,001 | up |
| ABCB1 | - | - | - | 6875,0 | 0,005 | up | 6606,3 | 0,000 | up |
| ABCB4 | - | - | - | - | - | - | 10,3 | 0,034 | up |
| ABCD1 | 4,1 | 0,013 | up | - | - | - | 2,0 | 0,006 | up |
| ABCD3 | - | - | - | - | - | - | 2,1 | 0,011 | down |
| ADHFE1 | 4,5 | 0,002 | up | - | - | - | - | - | - |
| AKR1B10 | 8,7 | 0,030 | up | - | - | - | - | - | - |
| ALDH1A3 | - | - | - | 18,1 | 0,000 | up | 9,5 | 0,016 | up |
| ALDH3A2 | 2,3 | 0,028 | down | - | - | - | - | - | - |
| ALDH4A1 | - | - | - | - | - | - | 2,3 | 0,021 | up |
| ALDH6A1 | - | - | - | - | - | - | 3,5 | 0,006 | up |
| CDKN2D | - | - | - | 2,3 | 0,008 | up | - | - | - |
| CHST11 | 2,2 | 0,035 | down | - | - | - | - | - | - |
| CYP2J2 | - | - | - | 3,6 | 0,045 | down | - | - | - |
| CYP51A1 | - | - | - | - | - | - | 3,4 | 0,002 | up |
| DHFR | 2,0 | 0,038 | down | 2,2 | 0,039 | down | 2,7 | 0,050 | down |
| DHRS12 | - | - | - | - | - | - | 2,1 | 0,011 | up |
|  | **MX-1/D vs. MX-1** | | | **MX-1/T vs. MX-1** | | | **MX-1/TD vs. MX-1** | | |
|  | FC | p-value | Regulation | FC | p-value | Regulation | FC | p-value | Regulation |
| **Chemoresistance-related genes** | | | | | | | | | |
| DHRS13 | - | - | - | - | - | - | 2,1 | 0,002 | up |
| DHRS3 | 29,8 | 0,045 | up | 43,1 | 0,014 | up | 44,2 | 0,005 | up |
| DHRS4 | - | - | - | 271,2 | 0,002 | down | 101,7 | 0,023 | down |
| DHRS4L2 | - | - | - | 659,2 | 0,001 | down | 441,3 | 0,000 | down |
| HSD17B14 | - | - | - | - | - | - | 3,7 | 0,023 | up |
| NQO1 | - | - | - | - | - | - | 2,3 | 0,016 | down |
| PLGLB1 | 2,3 | 0,015 | up | - | - | - | - | - | - |
| PPARA | - | - | - | 5,3 | 0,038 | up | 5,0 | 0,006 | up |
| PRAM1 | 3,4 | 0,012 | up | - | - | - | - | - | - |
| SAT1 | - | - | - | - | - | - | 3,1 | 0,013 | up |
| SLC19A2 | 2,2 | 0,039 | down | - | - | - | - | - | - |
| SLC22A18 | - | - | - | 3,5 | 0,001 | up | 4,0 | 0,001 | up |
| SLC25A13 | - | - | - | 4,6 | 0,003 | down | 3,9 | 0,000 | down |
| SLC29A2 | 2,0 | 0,025 | down | - | - | - | - | - | - |
| SLC2A1 | 3,6 | 0,018 | up | 3,5 | 0,016 | up | 3,5 | 0,048 | up |
| SLC6A6 | 2,4 | 0,032 | down | - | - | - | 2,2 | 0,008 | down |
| SOD2 | 3,1 | 0,037 | down | - | - | - | - | - | - |
| SOD3 | - | - | - | - | - | - | 14,8 | 0,021 | up |
| UGT2B10 | 2,7 | 0,003 | down | - | - | - | - | - | - |
| UGT2B11 | 2,8 | 0,004 | down | - | - | - | - | - | - |
| UGT2B7 | 2,7 | 0,005 | down | - | - | - | - | - | - |
| UGT8 | - | - | - | - | - | - | 2,3 | 0,023 | down |
| XDH | - | - | - | - | - | - | 30,5 | 0,049 | up |
| XPC | 2,3 | 0,041 | down | - | - | - | - | - | - |
| **CSC-related genes** | | | | | | | | | |
| CXCL8 | 59,6 | 0,015 | up | 56,6 | 0,002 | up | 39,0 | 0,003 | up |
| DDR1 | - | - | - | 3,5 | 0,033 | up | 3,6 | 0,008 | up |
| DLL1 | - | - | - | 4,6 | 0,039 | up | 3,7 | 0,003 | up |
| FLOT2 | - | - | - | - | - | - | 3,7 | 0,035 | up |
|  | **MX-1/D vs. MX-1** | | | **MX-1/T vs. MX-1** | | | **MX-1/TD vs. MX-1** | | |
|  | FC | p-value | Regulation | FC | p-value | Regulation | FC | p-value | Regulation |
| **CSC-related genes** | | | | | | | | | |
| FZD7 | - | - | - | 3,3 | 0,002 | up | 2,8 | 0,034 | up |
| JAG1 | - | - | - | - | - | - | 6,5 | 0,006 | up |
| MUC1 | - | - | - | - | - | - | 4,2 | 0,041 | up |
| **Immune response-related genes** | | | | | | | | | |
| CSF3 | - | - | - | 20,0 | 0,047 | up | 24,3 | 0,018 | up |
| CXCL2 | - | - | - | - | - | - | 22,8 | 0,034 | up |
| CXCL5 | - | - | - | - | - | - | 2,3 | 0,044 | down |
| IL17RC | - | - | - | - | - | - | 2,0 | 0,013 | up |
| IL18R1 | - | - | - | 4,7 | 0,036 | up | - | - | - |
| IL1B | - | - | - | 260,7 | 0,006 | up | 46,3 | 0,042 | up |
| IL6 | - | - | - | 265,1 | 0,013 | up | - | - | - |
| LTBP1 | - | - | - | - | - | - | 2,9 | 0,023 | up |
| LTBP3 | - | - | - | - | - | - | 4,4 | 0,039 | up |
| MYD88 | - | - | - | - | - | - | 3,0 | 0,045 | down |
| NLRP3 | - | - | - | 37,5 | 0,011 | up | - | - | - |
| OSMR | - | - | - | - | - | - | 2,2 | 0,023 | up |
| TICAM1 | - | - | - | - | - | - | 2,0 | 0,028 | up |
| TLR3 | - | - | - | 2,6 | 0,011 | down | - | - | - |
| **Cell adhesion and motility-related genes** | | | | | | | | | |
| ARHGDIB | 117,7 | 0,048 | up | - | - | - | - | - | - |
| CAV1 | - | - | - | 2,0 | 0,035 | down | - | - | - |
| CLDN1 | - | - | - | 2,3 | 0,027 | down | - | - | - |
| CLDN12 | - | - | - | 3,1 | 0,038 | up | 3,6 | 0,002 | up |
| FGF20 | - | - | - | - | - | - | 4,3 | 0,024 | down |
| GJA1 | - | - | - | 67,4 | 0,029 | down | - | - | - |
| GJC2 | - | - | - | 2,1 | 0,009 | down | - | - | - |
| GP1BB | 4,4 | 0,009 | up | 3,6 | 0,021 | up | 5,2 | 0,008 | up |
| ITGA3 | - | - | - | - | - | - | 2,3 | 0,004 | up |
| ITGB3 | - | - | - | - | - | - | 2,1 | 0,006 | up |
|  | **MX-1/D vs. MX-1** | | | **MX-1/T vs. MX-1** | | | **MX-1/TD vs. MX-1** | | |
|  | FC | p-value | Regulation | FC | p-value | Regulation | FC | p-value | Regulation |
| **Cell adhesion and motility-related genes** | | | | | | | | | |
| LAMB1 | - | - | - | 2,7 | 0,018 | down | 2,5 | 0,001 | down |
| LAMB2 | - | - | - | - | - | - | 2,2 | 0,011 | up |
| LAMC1 | - | - | - | 3,8 | 0,006 | up | - | - | - |
| MMP14 | 3,1 | 0,019 | up | 6,4 | 0,040 | up | - | - | - |
| MMP24 | - | - | - | 2,0 | 0,013 | up | - | - | - |
| PCDHB11 | 10,5 | 0,008 | up | 11,7 | 0,021 | up | 11,5 | 0,002 | up |
| PCDHB14 | 8,8 | 0,024 | up | 17,5 | 0,039 | up | 17,1 | 0,003 | up |
| PCDHB16 | 7,1 | 0,007 | up | - | - | - | - | - | - |
| PCDHB2 | 18,6 | 0,043 | up | - | - | - | - | - | - |
| PCDHB9 | 4,3 | 0,048 | up | - | - | - | - | - | - |
| PCDHGA2 | - | - | - | - | - | - | 2,9 | 0,016 | up |
| PCDHGA8 | - | - | - | 15,7 | 0,022 | up | 19,8 | 0,001 | up |
| PCDHGB7 | 2,6 | 0,001 | up | 5,1 | 0,011 | up | 6,4 | 0,000 | up |
| PTEN | 2,1 | 0,009 | down | - | - | - | - | - | - |
| PTK2B | - | - | - | - | - | - | 3,6 | 0,042 | down |
| RASA1 | - | - | - | 6,6 | 0,002 | up | - | - | - |
| RHOB | 2,1 | 0,010 | down | 4,7 | 0,003 | down | - | - | - |
| RHOF | - | - | - | - | - | - | 3,4 | 0,032 | up |
| RHOV | - | - | - | 2,6 | 0,012 | up | 2,1 | 0,041 | up |
| RND3 | - | - | - | - | - | - | 2,5 | 0,002 | down |
| SH3PXD2A | - | - | - | 12,2 | 0,014 | up | 9,4 | 0,038 | up |
| TLN1 | - | - | - | - | - | - | 3,0 | 0,030 | down |
| **Epigenetic regulators** | | | | | | | | | |
| CENPI | 2,3 | 0,003 | down | - | - | - | - | - | - |
| CENPM | 2,1 | 0,026 | down | - | - | - | - | - | - |
| DNMT1 | - | - | - | - | - | - | 2,1 | 0,019 | down |
| DNMT3A | - | - | - | - | - | - | 2,2 | 0,015 | down |
| H2AFY2 | 68,1 | 0,027 | down | - | - | - | - | - | - |
| HIST1H1C | 2,1 | 0,035 | up | - | - | - | - | - | - |
|  | **MX-1/D vs. MX-1** | | | **MX-1/T vs. MX-1** | | | **MX-1/TD vs. MX-1** | | |
|  | FC | p-value | Regulation | FC | p-value | Regulation | FC | p-value | Regulation |
| **Epigenetic regulators** | | | | | | | | | |
| HIST1H2AA | - | - | - | - | - | - | 2,4 | 0,026 | down |
| HIST1H2AB | - | - | - | - | - | - | 2,1 | 0,023 | down |
| HIST1H2AC | 22,1 | 0,047 | up | - | - | - | - | - | - |
| HIST1H2AD | - | - | - | - | - | - | 2,0 | 0,003 | down |
| HIST1H2AE | - | - | - | - | - | - | 2,1 | 0,004 | down |
| HIST1H2AG | 7,4 | 0,016 | up | - | - | - | - | - | - |
| HIST1H2AH | - | - | - | - | - | - | 2,2 | 0,008 | down |
| HIST1H2AI | - | - | - | - | - | - | 2,8 | 0,001 | down |
| HIST1H2AK | - | - | - | - | - | - | 2,1 | 0,005 | down |
| HIST1H2AL | - | - | - | - | - | - | 2,9 | 0,015 | down |
| HIST1H2BF | - | - | - | - | - | - | 2,3 | 0,007 | down |
| HIST1H2BH | - | - | - | - | - | - | 2,2 | 0,002 | down |
| HIST1H2BK | 13,6 | 0,001 | up | - | - | - | - | - | - |
| HIST1H2BN | - | - | - | - | - | - | 2,4 | 0,015 | down |
| HIST1H2BO | - | - | - | - | - | - | 2,2 | 0,002 | down |
| HIST1H3H | - | - | - | - | - | - | 2,3 | 0,002 | down |
| HIST1H3J | - | - | - | - | - | - | 2,8 | 0,036 | down |
| HIST1H4A | - | - | - | - | - | - | 2,1 | 0,031 | down |
| HIST1H4C | - | - | - | - | - | - | 2,0 | 0,011 | down |
| HIST1H4D | - | - | - | - | - | - | 2,6 | 0,010 | down |
| HIST1H4H | 19,8 | 0,018 | up | - | - | - | - | - | - |
| HIST1H4L | 3,3 | 0,030 | down | - | - | - | - | - | - |
| HIST2H2AA | 16,3 | 0,006 | up | - | - | - | - | - | - |
| HIST2H2BE | 16,1 | 0,021 | up | 2,5 | 0,008 | down | - | - | - |
| HIST3H2BB | 3,5 | 0,010 | down | - | - | - | - | - | - |
| HIST4H4 | 3,6 | 0,028 | down | - | - | - | 2,4 | 0,018 | down |
| KDM5B | - | - | - | - | - | - | 2,3 | 0,014 | up |
| KDM6B | - | - | - | - | - | - | 2,5 | 0,003 | up |
| METTL12 | 2,1 | 0,026 | down | - | - | - | - | - | - |
|  | **MX-1/D vs. MX-1** | | | **MX-1/T vs. MX-1** | | | **MX-1/TD vs. MX-1** | | |
|  | FC | p-value | Regulation | FC | p-value | Regulation | FC | p-value | Regulation |
| **Epigenetic regulators** | | | | | | | | | |
| PCGF5 | 2,7 | 0,042 | down | - | - | - | - | - | - |
| PCGF6 | 2,1 | 0,002 | down | - | - | - | - | - | - |
| PHF1 | - | - | - | - | - | - | 2,1 | 0,013 | up |
| PRMT2 | - | - | - | - | - | - | 2,0 | 0,000 | up |
| PRMT7 | 2,0 | 0,003 | down | - | - | - | - | - | - |
| RYBP | 2,0 | 0,001 | down | - | - | - | - | - | - |
| SMARCC1 | 2,4 | 0,008 | down | - | - | - | - | - | - |
| USP11 | - | - | - | - | - | - | 2,6 | 0,044 | up |
| **Channels and transporters** | | | | | | | | | |
| ABCB9 | - | - | - | - | - | - | 4,3 | 0,036 | up |
| ABCG4 | - | - | - | - | - | - | 5,5 | 0,018 | up |
| ATP13A2 | - | - | - | - | - | - | 2,3 | 0,012 | down |
| ATP2A1 | 2,9 | 0,040 | up | - | - | - | - | - | - |
| ATP2B4 | 9,2 | 0,048 | up | 12,8 | 0,017 | up | 7,6 | 0,040 | up |
| ATP5D | 2,5 | 0,000 | down | - | - | - | - | - | - |
| ATP5E | 3,1 | 0,034 | down | 2,2 | 0,043 | down | - | - | - |
| ATP5G1 | 2,3 | 0,039 | down | - | - | - | - | - | - |
| ATP5J2 | - | - | - | 2,3 | 0,037 | down | - | - | - |
| ATP6V0A4 | - | - | - | 4,8 | 0,004 | down | 2,8 | 0,047 | down |
| ATP8A2 | - | - | - | 17,5 | 0,017 | up | - | - | - |
| ATP8B3 | - | - | - | 7,6 | 0,020 | up | 7,9 | 0,001 | up |
| ATP9A | - | - | - | 2,6 | 0,049 | up | - | - | - |
| CACFD1 | - | - | - | - | - | - | 2,4 | 0,009 | up |
| CACNA2D1 | - | - | - | 27,4 | 0,019 | down | 16,3 | 0,001 | down |
| CACNA2D3 | - | - | - | - | - | - | 6,6 | 0,003 | down |
| CACNB3 | - | - | - | 2,3 | 0,017 | up | 2,4 | 0,001 | up |
| CACNG7 | 2,8 | 0,048 | up | 4,6 | 0,000 | up | 4,5 | 0,000 | up |
| CLCC1 | - | - | - | 2,0 | 0,010 | down | - | - | - |
| CLCN5 | 2,8 | 0,022 | down | - | - | - | - | - | - |
|  | **MX-1/D vs. MX-1** | | | **MX-1/T vs. MX-1** | | | **MX-1/TD vs. MX-1** | | |
|  | FC | p-value | Regulation | FC | p-value | Regulation | FC | p-value | Regulation |
| **Channels and transporters** | | | | | | | | | |
| CLNS1A | 2,1 | 0,045 | down | - | - | - | - | - | - |
| GABRB1 | - | - | - | - | - | - | 2,1 | 0,008 | down |
| GRIN2C | 5,0 | 0,017 | up | - | - | - | - | - | - |
| KCNA6 | - | - | - | 48,0 | 0,017 | up | - | - | - |
| KCNAB2 | - | - | - | 5,9 | 0,042 | up | 3,9 | 0,010 | up |
| KCND2 | - | - | - | 28,0 | 0,041 | down | - | - | - |
| KCNF1 | 3,2 | 0,012 | up | 30,6 | 0,005 | down | 21,0 | 0,035 | down |
| KCNMA1 | - | - | - | - | - | - | 6,0 | 0,036 | up |
| KCNN4 | - | - | - | 2,5 | 0,037 | up | 2,6 | 0,003 | up |
| KCNT1 | - | - | - | - | - | - | 2,0 | 0,049 | down |
| KCTD14 | - | - | - | 2,5 | 0,032 | down | 2,4 | 0,003 | down |
| MCOLN3 | 3,7 | 0,030 | up | - | - | - | - | - | - |
| P2RX4 | - | - | - | - | - | - | 2,1 | 0,019 | up |
| SLC12A2 | 2,0 | 0,022 | down | - | - | - | - | - | - |
| SLC16A13 | - | - | - | - | - | - | 2,0 | 0,030 | up |
| SLC18B1 | 2,5 | 0,042 | down | - | - | - | - | - | - |
| SLC19A2 | 2,2 | 0,039 | down | - | - | - | - | - | - |
| SLC22A18 | - | - | - | 3,5 | 0,001 | up | 4,0 | 0,001 | up |
| SLC25A13 | - | - | - | 4,6 | 0,003 | down | 3,9 | 0,000 | down |
| SLC25A15 | 2,1 | 0,024 | down | - | - | - | - | - | - |
| SLC25A34 | - | - | - | - | - | - | 2,6 | 0,007 | up |
| SLC25A40 | - | - | - | 18,8 | 0,010 | up | 14,8 | 0,001 | up |
| SLC26A2 | - | - | - | 3,4 | 0,010 | up | - | - | - |
| SLC29A2 | 2,0 | 0,025 | down | - | - | - | - | - | - |
| SLC30A4 | - | - | - | 2,1 | 0,046 | up | 2,1 | 0,002 | up |
| SLC30A6 | - | - | - | 2,1 | 0,039 | down | - | - | - |
| SLC35E2 | - | - | - | - | - | - | 2,1 | 0,012 | up |
| SLC35E3 | - | - | - | - | - | - | 3,2 | 0,016 | up |
| SLC35F2 | - | - | - | 2,6 | 0,024 | down | - | - | - |
|  | **MX-1/D vs. MX-1** | | | **MX-1/T vs. MX-1** | | | **MX-1/TD vs. MX-1** | | |
|  | FC | p-value | Regulation | FC | p-value | Regulation | FC | p-value | Regulation |
| **Channels and transporters** | | | | | | | | | |
| SLC35G1 | - | - | - | - | - | - | 2,2 | 0,008 | down |
| SLC37A1 | 2,1 | 0,007 | down | - | - | - | - | - | - |
| SLC37A2 | - | - | - | 22,1 | 0,015 | up | 12,4 | 0,012 | up |
| SLC39A8 | - | - | - | 125,2 | 0,002 | down | 60,1 | 0,000 | down |
| SLC4A11 | - | - | - | 4,3 | 0,022 | up | 3,6 | 0,014 | up |
| SLC4A3 | - | - | - | - | - | - | 2,3 | 0,019 | up |
| SLC4A8 | - | - | - | 4,2 | 0,014 | up | 4,2 | 0,018 | up |
| SLC5A12 | 2,6 | 0,001 | up | - | - | - | - | - | - |
| SLC6A2 | - | - | - | - | - | - | 2,4 | 0,036 | down |
| SLC6A6 | 2,4 | 0,032 | down | - | - | - | 2,2 | 0,008 | down |
| SLC6A8 | 2,2 | 0,045 | up | - | - | - | 2,6 | 0,014 | up |
| SLC9A7 | - | - | - | 3,4 | 0,000 | up | 2,9 | 0,001 | up |
| TPCN1 | - | - | - | 2,1 | 0,007 | up | 2,0 | 0,044 | up |
| TRPC6 | - | - | - | - | - | - | 2,8 | 0,049 | down |
| TRPM4 | - | - | - | 2,5 | 0,027 | up | 2,8 | 0,000 | up |
